# Zero Calcium Score as a Filter for Further Testing in Patients Admitted to the Coronary Care Unit with Chest Pain

**DOI:** 10.5935/abc.20170076

**Published:** 2017-08

**Authors:** Luis Cláudio Lemos Correia, Fábio P. Esteves, Manuela Carvalhal, Thiago Menezes Barbosa de Souza, Nicole de Sá, Vitor Calixto de Almeida Correia, Felipe Kalil Beirão Alexandre, Fernanda Lopes, Felipe Ferreira, Márcia Noya-Rabelo

**Affiliations:** 1 Escola Bahiana de Medicina e Saúde Pública, Salvador, BA - Brazil; 2 Emory University, Georgia - USA

**Keywords:** Acute Coronary Syndrome / diagnosis, Chest Pain, Calcinosis / diagnosis, Predictive Value of Tests, Emergency Medical Services

## Abstract

**Background:**

The accuracy of zero coronary calcium score as a filter in patients with chest pain has been demonstrated at the emergency room and outpatient clinics, populations with low prevalence of coronary artery disease (CAD).

**Objective:**

To test the gatekeeping role of zero calcium score in patients with chest pain admitted to the coronary care unit (CCU), where the pretest probability of CAD is higher than that of other populations.

**Methods:**

Patients underwent computed tomography for calcium scoring, and obstructive CAD was defined by a minimum 70% stenosis on invasive angiography.

**Results:**

In 146 patients studied, the prevalence of CAD was 41%. A zero calcium score was present in 35% of the patients. The sensitivity and specificity of zero calcium score yielded a negative likelihood ratio of 0.16. After logistic regression adjustment for pretest probability, zero calcium score was independently associated with lower odds of CAD (OR = 0.12, 95%CI = 0.04-0.36), increasing the area under the ROC curve of the clinical model from 0.76 to 0.82 (p = 0.006). Zero calcium score provided a net reclassification improvement of 0.20 (p = 0.0018) over the clinical model when using a pretest probability threshold of 10% for discharging without further testing. In patients with pretest probability < 50%, zero calcium score had a negative predictive value of 95% (95%CI = 83%-99%), with a number needed to test of 2.1 for obtaining one additional discharge.

**Conclusion:**

Zero calcium score substantially reduces the pretest probability of obstructive CAD in patients admitted to the CCU with acute chest pain. (Arq Bras Cardiol. 2017; [online].ahead print, PP.0-0)

## Introduction

The majority of patients presenting to the hospital with acute chest pain do not have obstructive coronary artery disease (CAD). These patients often undergo imaging tests before discharge.^1^ The efficiency of this strategy is challenged by the low yield for positive results.^2^ Furthermore, many imaging tests are only available in business hours, are time-consuming and costly, have several contraindications and require expert interpretation. For example, computed tomography (CT) coronary angiography should be avoided in patients with renal failure or known allergies to dye; functional tests require pharmacological or physical stress and are usually not available 24 hours a day.

A filter is a simple test where a negative result obviates the need for more complex tests. Previous studies have suggested that zero calcium score has a sufficiently low negative likelihood ratio to play a filtering role in patients with chest pain.^3^ However, these studies have focused on patients presenting to the emergency room and the outpatient clinic, a population with low prevalence of CAD.

Our aim was to study the diagnostic performance of zero calcium score as a filter for other imaging tests in patients with acute chest pain admitted to the coronary care unit (CCU) of a tertiary-care hospital, where the prevalence of disease is expected to be at least intermediate. We explored the negative predictive value of zero calcium score in the whole group and according to strata of pretest probability.

## Methods

### Sample selection

Between September 2011 and October 2013, consecutive patients admitted to the CCU of a tertiary-care hospital were asked to participate in the Chest Pain Registry, a prospective, and observational study. Among 370 patients included in the Registry, a subgroup of 146 underwent coronary calcium scoring based on the following entry criteria: at least 18 years of age, no implanted coronary stents, no coronary artery bypass grafts and willingness to sign a written informed consent. Of those who did not undergo calcium scoring, 71 had coronary stents, 24 had previous coronary artery bypass graft surgery and 129 refused radiation exposure.

### Coronary calcium score

All patients were imaged with a commercially available 64-multidetector CT scanner (Aquilion, Toshiba Medical Systems, Tochigi, Japan). The scans were obtained using slice collimation 4 x 3.0 mm, 300 mA, 120 kV and gantry rotation time 0.4 s. Calcium scoring using the Agatston method was performed in remote workstations (Vitrea2 Version 3.0.9.1, Vital Images, Minnetonka, Minnesota). A sole radiologist unaware of patient's characteristics or presence of obstructive CAD scored all scans. Calcium scoring was performed within a week of other noninvasive imaging and invasive coronary angiography.

### Obstructive CAD

Patients underwent invasive coronary angiography or a provocative noninvasive test (perfusion magnetic resonance imaging or nuclear single-photon emission computed tomography) at the discretion of the cardiologist. Invasive angiography was performed whenever the ischemic defect size was ≥ 5% of the left ventricular myocardium on noninvasive imaging. The readers of invasive and noninvasive images were blinded to the calcium score. Obstructive CAD was defined as luminal stenosis ≥ 70% by invasive angiography. A normal or mildly abnormal noninvasive test, defined as ischemic defect size < 5% of left ventricular myocardium, was interpreted as negative for obstructive CAD and no further testing was required. Patients were classified as not having obstructive CAD if one of the following dominant diagnosis was confirmed by imaging: pericarditis, pulmonary embolism or aortic dissection.

### Pretest probability of obstructive CAD

The entire cohort of 370 patients was used to generate a multivariate clinical model for predicting pretest probability of CAD based on admission data. Three sets of variables were studied as potential predictors for obstructive CAD: 13 variables of medical history, 14 characteristics of chest discomfort and 8 variables related to physical examination and laboratory tests. These included ischemia on electrocardiogram, positive troponin, physical and radiographic signs of left ventricular failure, N-Terminal Pro-B-Type Natriuretic Peptide (NT-proBNP, enzyme-linked fluorescent assay, Biomérieux, France), high-sensitivity C-reactive protein (CRP, nephelometry, Dade-Behring, USA), white cell count, plasma glucose and hemoglobin. All serum specimens were collected at presentation to the emergency room. All variables, in case of normal distribution, were presented through mean and standard deviation and, in the case of non-normal distribution, were presented through median and interquartile range. By multivariate logistic regression analysis, variables remained independent predictors: age, male gender, chest pain relief with nitrate, signs of heart failure, ischemia on electrocardiogram and positive troponin. For discrimination of obstructive CAD, the area under the curve of this clinical model was 0.80 [95% confidence interval (CI) = 0.75-0.84] and calibration by Hosmer-Lemeshow's test led to a χ2 = 1.95 (p = 0.98). This was the reference model used to evaluate the incremental value of the calcium score and to define the pretest probability for the sensitivity analysis of the predictive value of calcium scoring.

### Statistical analysis

The sample size was calculated to provide a maximum error of ± 12% for 95% CI of sensitivity and specificity. Assuming a sensitivity of 90% and a specificity of 50%, 25 patients with and 67 patients without obstructive CAD were required to provide this estimate precision. Anticipating a CAD prevalence of 50%, at least 134 patients had to be enrolled in the study.

A negative calcium score was defined as zero, while a positive score was defined as anything other than zero. Based on this predefined cut-off, sensitivity, specificity, positive and negative likelihood ratios were described as measures of accuracy with 95% CI. The incremental predictive value of zero calcium score over the pretest probability model was tested by comparing the area under the curve of this model versus the area under the curve of a second model containing clinical and binary calcium score information. This second model was derived from logistic regression analysis.

The accuracy of zero calcium score to reclassify the clinical model pretest probability to < 10% was evaluated by Pencina's method of net reclassification improvement (NRI).^4^

Finally, negative predictive values and number needed to test for discharging one additional patient were reported in the entire group and in subgroups of pretest probability < 50% or ≥ 50%. The same analysis was done in the groups of normal electrocardiogram and troponin versus either one of these tests abnormal.

The SPSS Statistical Software (Version 21.0, SPSS Inc., Chicago, Illinois, USA) was used for data analysis, and final statistical significance was defined as p < 0.05 in all cases.

## Results

### Sample characteristics

One hundred forty-six patients with acute chest pain were studied, aged 59 ± 16 years, 56% males. Ischemic electrocardiographic changes were present in 56% of patients, 42% had positive troponin and 71% had at least one of these two tests abnormal. Obstructive CAD was present in 60 patients (prevalence of 41%) and all cases were confirmed by invasive angiography. Among 86 patients without obstructive CAD, 28 had invasive angiography and 58 were deemed not to have obstructive CAD by noninvasive imaging only. The final diagnosis in patients without CAD was pericarditis (8), dyspepsia (4), muscular pain, pneumonia and pulmonary embolism (one each). The remaining 71 patients had chest pain of unclear etiology. Clinical and laboratory characteristics are depicted in Table 1.

**Table 1:** Clinical and laboratory characteristics

Sample Size	146
Age (years)	59 ± 16
Male Gender	82 (56%)
Ischemic EKG	81 (55%)
Positive troponin	61 (42%)
CAD history	35 (24%)
Diabetes	43 (29%)
Systemic hypertension	108 (74%)
Smoking	19 (13%)
Total cholesterol (mg/dl)	183 ± 59
LDL-cholesterol (mg/dl)	112 ± 64
HDL-cholesterol (mg/dl)	43 ± 15
Triglycerides (mg/dl)	165 ± 152
Creatinine (mg/dl)	1.08 ± 0.85
Calcium score (Agatston)	66 (0 – 722)
Zero calcium score	51 (35%)
Obstructive CAD	60 (41%)

EKG: electrocardiogram; CAD: coronary artery disease; Ischemic EKG: T-wave inversion or ST-segment deviation. Positive troponin: troponin change to a level beyond the 99th percentile. Calcium score is described as median and interquartile range, and the remaining numeric variables as mean and standard deviation.

### Diagnostic accuracy of zero calcium score

Calcium score had a non-normal distribution, with a median of 66 (interquartile range = 0-722). Zero calcium score was seen in 35% of patients. Among 60 patients with obstructive CAD, 55 had calcium score > zero, yielding a sensitivity of 92% (95% CI = 81%-97%). Among 86 patients without obstructive CAD, 46 had zero calcium score, specificity of 54% (95% CI = 43%-64%). The observed accuracy yielded a good negative likelihood ratio of 0.16 (95% CI = 0.07-0.37) and a poor positive likelihood ratio of 1.97 (95% CI = 1.55-2.50).

### Incremental diagnostic value of zero calcium score

**Figure 1:**
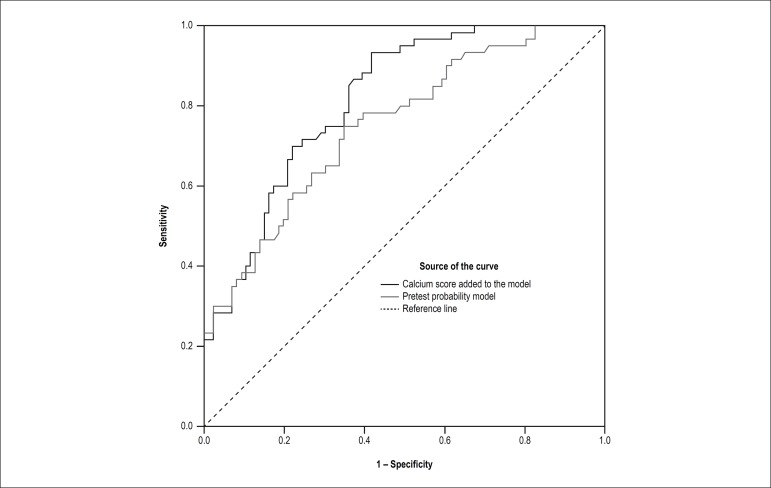
Incremental value of zero calcium score added to the reference model of pretest probability. The area under the curve increased from 0.76 to 0.82 (p = 0.006).

After adjustment for pretest probability based on the clinical model (OR = 1.04; 95% CI = 1.02-1.06; p < 0.001), zero calcium score was independently associated with absent CAD (OR = 0.12; 95% IC = 0.04-0.34; p < 0.001). The prediction based on the clinical model had an area under the curve of 0.76 (95% CI = 0.67-0.83), which improved to 0.82 (95% CI = 0.75-0.88; p = 0.006) when calcium scoring was added to the curve (Figure 1).

### Net reclassification by calcium score

The target for theoretical discharge based on the clinical model with no further testing (CAD probability < 10%) was present in only 8.2% of patients. Upon inclusion of zero calcium score in the model, 23% of patients were classified as < 10% probability of CAD. Twenty-six of 86 patients without obstructive CAD were correctly reclassified to < 10% probability and 3 were incorrectly reclassified to ≥ 10%. Thus, the NRI for individuals with no CAD was 0.23 (p = 0.0001). Among 60 patients with CAD, none were correctly up-reclassified and 2 were incorrectly reclassified to < 10% probability.

The NRI of patients with CAD was -0.03 (p = 0.16). As a result, the overall NRI was 0.20 (p = 0.0018), indicating a reasonable proportion of patients with no CAD reclassified to < 10% probability (Table 2).

**Table 2:** Net reclassification (NRI) of low or high probability (cut-off 10%) according to zero calcium score.

CAD Status	Probability Threshold (10%)	Reclassification by Ca Score		NRI	Z Score	p Value
Present = 60		Low	High			
	Low = 0	--	--	- 0.03	1.41	0.16
	High = 60	2	58			
Absent = 86						
	Low = 12	9	3	+ 0.23	3.92	< 0.001
	High = 74	23	51			
			Global NRI	+ 0.20	3.12	0.002

CAD: coronary artery disease.

### Sensitivity analysis of negative predictive values by pretest probability

The overall negative predictive value of zero calcium score for obstructive CAD was 90% (95% CI = 78%-96%). Ninety-two patients (63%) had a pretest probability < 50% with a disease prevalence of 27%. In this group, 43 patients had a zero calcium score, with a negative predictive value of 95% (95% CI = 83%-99%). Since 47% of the patients had zero calcium score, the number needed to test for obtaining one additional discharge (< 10% probability) was 2.1. In this group, calcium score had a high yield for a negative result.

On the other hand, 54 patients (37%) had a pretest probability of CAD ≥ 50% with a disease prevalence of 65%. In this group, only 8 patients (15%) had a zero calcium score, with a negative predictive value of 63% (95% CI = 23%-90%). In this group of high pretest probability, zero calcium score had a low yield for a negative result (Table 3).

**Table 3:** Negative predictive value and number needed to test for one additional discharge, according to pretest probability group and electrocardiogram/troponin results

	Sample	CAD Prevalence	Negative Predictive Value	Number Needed to Test
Pretest Probability < 50%	92	27%	95%	2.1
Pretest Probability ≥ 50%	54	65%	63%	6.6
Normal EKG and troponin	42	21%	100%	2.0
Positive EKG or troponin	104	49%	83%	2.4

Number needed to test: to avoid one further complex test. EKG: electrocardiogram; CAD: coronary artery disease.

Forty-two patients had normal electrocardiogram and troponin with CAD prevalence of 21%. Half of them had zero calcium score (number needed to test = 2), providing a negative predictive value of 100%. Of the remaining patients with either ischemic changes on electrocardiogram or positive troponin (CAD prevalence of 49%), 41% had zero calcium score but the negative predictive value was only 83% (95% CI = 70%-97%) (Table 3).

## Discussion

The present study extends the validation of zero calcium score as a filter for further diagnostic testing to patients with acute chest pain admitted to the CCU. It is important to emphasize that our target population are individuals with an intermediate pretest probability of CAD, having undergone an initial clinical judgment in the emergency room. Usually, these patients perform provocative tests of coronary ischemia or CT coronary angiography. In this context, the calcium score could be used as a filter to perform more complex tests. The relatively high prevalence of coronary disease in this setting raises concern regarding the negative predictive value of the test. In fact, we found that 41% of patients had obstructive CAD. Since the prevalence of zero calcium score was 35%, roughly 3 patients had to undergo calcium scoring to avoid one additional diagnostic test. In addition, calcium score increased the accuracy of a clinical model of pretest probability by improving the area under the curve and net reclassifying 23% of patients from high to low probability of disease.

We performed a sensitivity analysis to identify the subgroup better suited to calcium scoring according to the pretest probability of CAD. We suggest that if the pretest probability is less than 50%, a zero calcium score has a 95% negative predictive value. For every two patients tested, one would be discharged without the need for further testing. In the subgroup of normal electrocardiogram and negative troponin, the negative predictive value was 100%. Despite a general low case-fatality rate, there were no deaths in the group of zero calcium score.

Clinical interpretation of our findings suggests that there is a role for calcium scoring as a filter for other diagnostic tests in patients admitted to the CCU, provided the pretest probability for CAD is not high. However, our study brings initial data that need to be better tested in practice. A well-established filter for a potentially serious condition is the use of D-dimer in patients with low-to-intermediate probability of pulmonary embolism. D-dimer has a negative likelihood ratio of 0.13,^5^ which is very similar to what we found in patients with suspected CAD and zero calcium score. Patients with low-to-intermediate probability of pulmonary embolism and a negative D-dimer comprise 24% of patients with suspected pulmonary embolism.^5^ In our study, patients with CAD probability < 50% and zero calcium score comprised 29% of patients. The similarities between D-dimer (as a rule-out test for pulmonary embolism) and calcium scoring (as a rule-out test for CAD) highlight the potential of this approach in acute chest pain patients. One might be tempted to go directly to CT coronary angiography, instead of filtering it with a calcium score. While CT angiography is an option in some imaging centers, a few points should be addressed. First, CT angiography is not available 24/7 in most hospitals because it requires medical expertise for interpretation. The binary interpretation of coronary calcium on CT is simple and demands minimal training.

Second, there are technical difficulties and contraindications to CT angiography, which in the ROMICAT Study prevented this test in 1270 of 1869 (68%) patients with acute chest pain.^6^ Third, despite a much better positive likelihood ratio of CT angiography, its negative likelihood ratio is very similar to zero calcium score. In the CORE-64 trial, the negative likelihood ratio of CT angiography was 0.19.^7^ A reasonable approach would be to discharge patients with pretest probability < 50% and a zero calcium score. Patients with a positive calcium score would undergo CT angiography. This algorithm would not only reduce the time spent in the hospital to rule-out CAD, but also reduce costs and complications from more complex tests.

The diagnostic performance of zero calcium score described in the present study is in line with previous articles that reported good negative likelihood ratios and negative predictive values in emergency department patients.^8-12^ However, their good negative predictive values were in part the result of a low pretest probability of disease. Our uniqueness relies on the study of patients admitted to the CCU of a tertiary-care hospital with a much higher prevalence of disease. We demonstrated a reasonable negative predictive value in this population, extending the findings already reported in emergency room patients to the CCU. Zero calcium score can be used to exclude obstructive CAD in patients with low-to-intermediate (< 50%) probability based on sensitivity analysis.

### Limitation

The limitation of our work is a relatively small sample size, which provided only moderate precision according to our confidence intervals. Therefore, future studies should confirm our point-estimates of accuracy and predictive values. From the point of view of reliability of the scientific data, ideally, all patients should have undergone invasive coronary angiography. All patients labeled as positive for obstructive CAD had confirmation by invasive angiography, but most labeled as negative for obstructive disease had only non-invasive imaging.

## Conclusion

In conclusion, our study suggests that the use of zero calcium score substantially reduces pretest probability of obstructive CAD in patients admitted to the CCU with acute chest pain.
